# Ethyl­enediammonium bis­(5-methyl-3-oxo-2-phenyl-2,3-dihydro­pyrazol-1-ide): a hydrogen-bond-supported supra­molecular ionic assembly

**DOI:** 10.1107/S1600536808026378

**Published:** 2008-08-23

**Authors:** Ruibo Xu, Xingyou Xu, Daqi Wang, Xujie Yang, Xin Wang

**Affiliations:** a Materials Chemistry Laboratory, Nanjing University of Science and Technology, Nanjing 210094, People’s Republic of China; b Department of Chemical Engineering, Huaihai Institute of Technology, Lianyungang 222005, People’s Republic of China; c College of Chemistry and Chemical Engineering, Liaocheng University, Shandong 252059, People’s Republic of China

## Abstract

The title compound, C_2_H_10_N_2_^2+^·2C_10_H_9_N_2_O^−^, is composed of deprotonated 5-methyl-2-phenyl-1*H*-pyrazol-3(2*H*)-one anions (PMP^−^) and protonated ethyl­enediamine cations (H_2_en^2+^). The ethyl­enediammonium ion is located on a crystallographic inversion center. The dihedral angle between the phenyl and pyrazole rings is 39.73 (8)°. The two components are connected through N—H⋯O and N—H⋯N hydrogen bonds, forming an infinite three-dimensional network.

## Related literature

For related literature on pyrazolones, see: Cerchiaro *et al.* (2006 ▶). For conductivity data for ionic electrolytes, see: Kwak *et al.* (2004 ▶); Allmann *et al.* (1990 ▶). For background information on hydrogen bonds and their importance and applications, see: Fu *et al.* (2004 ▶); Hernández-Galindo *et al.* (2007 ▶); Hu *et al.* (2004 ▶); Li & Wang (2007 ▶); Peng *et al.* (2005 ▶); Yang *et al.* (2002 ▶, 2005 ▶, 2006 ▶); Zhou *et al.* (2006 ▶).
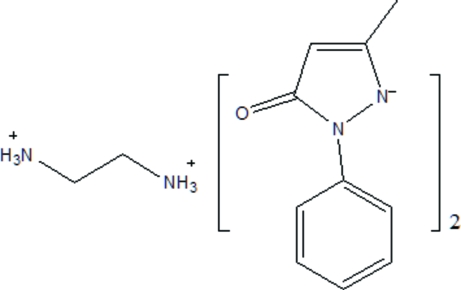


## Experimental

### 

#### Crystal data

C_2_H_10_N_2_^2+^·2C_10_H_9_N_2_O^−^
*M* *_r_* = 408.50
Tetragonal, 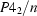

*a* = 17.179 (2) Å
*c* = 7.0929 (15) Å
*V* = 2093.2 (6) Å^3^
*Z* = 4
Mo *K*α radiation
μ = 0.09 mm^−1^
*T* = 298 (2) K
0.24 × 0.22 × 0.17 mm

#### Data collection

Siemens SMART CCD area-detector diffractometer
Absorption correction: none
10541 measured reflections
1856 independent reflections
1284 reflections with *I* > 2σ(*I*)
*R*_int_ = 0.045

#### Refinement

*R*[*F* ^2^ > 2σ(*F* ^2^)] = 0.043
*wR*(*F* ^2^) = 0.141
*S* = 1.04
1856 reflections
136 parameters
H-atom parameters constrained
Δρ_max_ = 0.22 e Å^−3^
Δρ_min_ = −0.16 e Å^−3^

### 

Data collection: *SMART* (Siemens, 1996 ▶); cell refinement: *SAINT* (Siemens, 1996 ▶); data reduction: *SAINT*; program(s) used to solve structure: *SHELXS97* (Sheldrick, 2008 ▶); program(s) used to refine structure: *SHELXL97* (Sheldrick, 2008 ▶); molecular graphics: *SHELXTL* (Sheldrick, 2008 ▶); software used to prepare material for publication: *SHELXTL*.

## Supplementary Material

Crystal structure: contains datablocks I, global. DOI: 10.1107/S1600536808026378/zl2127sup1.cif

Structure factors: contains datablocks I. DOI: 10.1107/S1600536808026378/zl2127Isup2.hkl

Additional supplementary materials:  crystallographic information; 3D view; checkCIF report

## Figures and Tables

**Table 1:** Hydrogen-bond geometry (Å, °)

*D*—H⋯*A*	*D*—H	H⋯*A*	*D*⋯*A*	*D*—H⋯*A*
N3—H3*A*⋯O1	0.89	1.94	2.743 (2)	149
N3—H3*B*⋯O1^i^	0.89	1.79	2.672 (2)	173
N3—H3*C*⋯N2^ii^	0.89	2.04	2.924 (3)	173

Symmetry codes: (i) 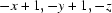; (ii) 
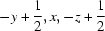
.

**Fig. 1.**
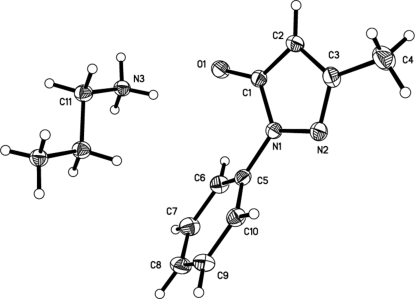
ORTEP drawing of the structure of the title compound with the atomic numbering scheme.

**Fig. 2.**
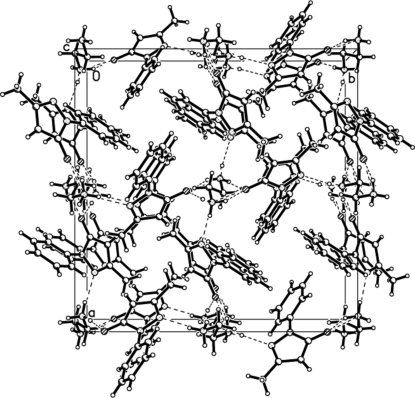
Stereoview and packing diagram for the title compound viewed along the c-axis.

**Fig. 3.**
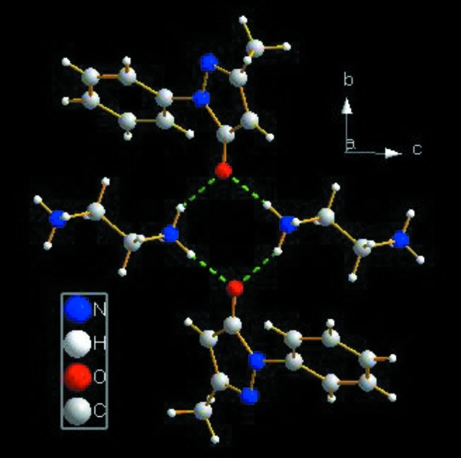
Part of the crystal structure of the title compound, showing the 8-membered rings formed by N—H···O interactions. Dashed lines indicate H-bonds.

**Fig. 4.**
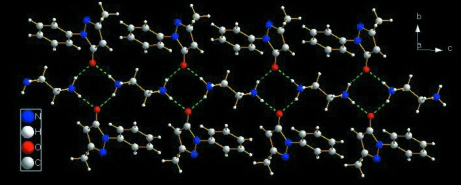
The crystal structure of title compound, showing the two-dimensional network parallel to the bc plane formed through N—H···O H-bond interactions. H-bonds are shown as dashed lines.

**Fig. 5.**
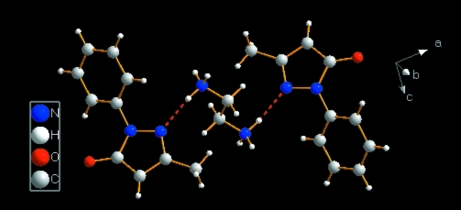
Part of the crystal structure of the title compound, showing the intermolecular N—H···N interactions. Dashed lines indicate H-bonds.

## supplementary crystallographic information

### Comment

Hydrogen bonds (H-bonds) are of importance in a large number of chemical and biological processes and in many practical applications (Li *et al.*, 2007; Peng *et al.*, 2005; Yang *et al.*, 2006; Yang *et al.*, 2002). Many interesting two- and three-dimensional frameworks have been designed and produced based on H-bonds (Fu *et al.*, 2004; Hu *et al.*, 2004; Zhou *et al.*, 2006). The chemistry of pyrazylone and its derivatives is particularly interesting because of their potential application in medicinal chemistry (Cerchiaro *et al.*, 2006), so 1-phenyl-3-methyl-pyrazole-5-one (PMP), which contains imino and carbonyl groups in its heterocycle, was choosen to react with the amino groups of ethylene diamine (en). The synthesis and crystal structure of the product of this reaction are reported here.

The title compound (Fig. 1) contains two crystallographically independent ions: the [H_2_en]^2+^ cation which has two cationic ammonium groups, and the [PMP]^-^ anion. In the [PMP]^-^ anion, the bond length of C(1)—O(1) is 1.288 (3) Å, much shorter than that of C—O (1.43 Å), but close to that of C═O (1.22 Å) (Yang *et al.*, 2005), indicating that the pyrazole-one ring of the title compound is present in the keto form, not the enol form. The N(2) atom of the deprotonated imino group of PMP is electron rich, therefore, the O atom of the carbonyl group and the N(2) atom of the pyrazole-one ring of the PMP molecule can be expected to be good hydrogen bond acceptors, while the H atoms of the [H_2_en]^2+^ ammonium cations are expected to be good hydrogen donors. The Jeffrey criterion for H-bonds used by the International Union of Crystallography defines the largest distance between the hydrogen and the acceptor atoms as 2.60 Å for H···O and 2.63 Å for H···N, respectively (Hernández-Galindo *et al.*, 2007). Compared to these data, the distances of H(3)···O(1) (-x+1, -y+1/2, -z) and H(3)···N(2)(x,-y+1/2, -z+1/2) for the title compound, respectively being 1.786 Å (or 1.940 Å) and 2.039 Å, are much shorter than the above-mentioned largest limit, providing a powerful evidence of the formation of H-bonds between these separate components.

There are no covalent interactions between the separate components of the title compound, but electrostatic and H-bonding interactions are present. All H atoms of the ammonium group, the O atom of the carbonyl group and the N atom of the deprotonated imino group in the title compound are engaged in intermolecular H-bonds which link the molecule into an extended three-dimensional network (Fig. 2). The H-bonds can be clearly seen in Fig. 2 to Fig. 5, and are summarized in Table 1. There are two kinds of H-bonds in the crystal structure: N—H···O and N—H···N. As shown in Fig. 3, the oxygen atom of the carbonyl group from [PMP]^-^ takes part in the H-bonding to the terminal nitrogen of [H_2_en]^2+^, forming intermolecular N—H···O H-bonds that result in the formation of 8-membered rings. Each of the two ammonium groups of the [H_2_en]^2+^ cation engages in these 8—membered rings, further linking the [H_2_en]^2+^ and [PMP]^-^ ions into a two-dimensional network *via* these N—H···O H-bonds (Fig. 4). It can be seen from Fig. 5 that the nitrogen atom of the deprotonated imino group of [PMP]^-^ takes part in the H-bonding to the terminal nitrogen of [H_2_en]^2+^, forming intermolecular N—H···N H-bonds. Therefore, through intermolecular N—H···O and N—H···N H-bonding interactions, each [PMP]^-^ anion is linked to three [H_2_en]^2+^ cations and each [H_2_en]^2+^ cation to six adjacent [PMP]^-^ anions. The two components thus construct a supramolecular assembly with a three-dimensional hydrogen bonded framework.

### Experimental

A solution of PMP (2 mmol in 10 ml anhydrous methanol) was added dropwise with constant stirring to the solution of en (2 mmol in 10 ml anhydrous methanol) at 323 K for 2 h. The resulting mixture was filtrated. After cooling, the filtrate was evaporated at ambient environment. Several days later, pink blocky crystals suitable for X-ray analysis were collected and washed with a small amount of methanol and dried at room temperature (yield 67%. m.p. 438–441 K). Anal. Calcd (%) for C_22_H_28_N_6_O_2_ (Mr = 408.5): C, 64.69; H, 6.91; N, 20.57. Found (%): C, 64.73; H, 6.97; N, 20.52. UV-vis (methanol): λ_max_ = 239 nm, ε = 2.655. The molar conductance of the compound in anhydrous methanol was 45.1 Ω^-1^cm^2^mol^-1^, much lower than that expected for a 1:2 electrolyte, indicating that the compound is forming ion pairs and larger assemblies tightly bonded to each other by e.g. hydrogen bonds (Allmann *et al.*, 1990; Kwak *et al.*, 2004).

### Refinement

H atoms were placed in calculated positions with C—H = 0.92Å (pyrazolyl), 0.93 Å (phenyl), 0.96 Å (methyl), 0.97 Å (methylene) and N—H = 0.89 Å (amino), and were refined in riding mode with *U*_iso_(H) = 1.5 *U*_eq_(C) (methyl) and *U*_iso_(H) = 1.2 *U*_eq_(C, N) (pyrazolyl, phenyl, methylene and amino).

### Crystal data

**Table:**

C_2_H_10_N_2_^2+^·2(C_10_H_9_N_2_O_1_^–^)	*F*_000_ = 872
*M**_r_* = 408.50	*D*_x_ = 1.296 Mg m^−^^3^
Tetragonal, *P*4_2_/*n*	Melting point = 441–438 K
*a* = 17.179 (2) Å	Mo *K*α radiation λ = 0.71073 Å
*b* = 17.179 (2) Å	Cell parameters from 2381 reflections
*c* = 7.0929 (15) Å	θ = 3.1–24.1º
α = 90º	µ = 0.09 mm^−^^1^
β = 90º	*T* = 298 (2) K
γ = 90º	Block, pink
*V* = 2093.2 (6) Å^3^	0.24 × 0.22 × 0.17 mm
*Z* = 4	

### Data collection

**Table:**

Siemens SMART CCD area-detector diffractometer	1284 reflections with *I* > 2σ(*I*)
Radiation source: fine-focus sealed tube	*R*_int_ = 0.046
Monochromator: graphite	θ_max_ = 25.0º
*T* = 298(2) K	θ_min_ = 1.7º
φ and ω scans	*h* = −18→20
Absorption correction: none	*k* = −15→20
10541 measured reflections	*l* = −8→8
1856 independent reflections	

### Refinement

**Table:**

Refinement on *F*^2^	Secondary atom site location: difference Fourier map
Least-squares matrix: full	Hydrogen site location: inferred from neighbouring sites
*R*[*F*^2^ > 2σ(*F*^2^)] = 0.043	H-atom parameters constrained
*wR*(*F*^2^) = 0.141	*w* = 1/[σ^2^(*F*_o_^2^) + (0.0642*P*)^2^ + 1.1449*P*] where *P* = (*F*_o_^2^ + 2*F*_c_^2^)/3
*S* = 1.04	(Δ/σ)_max_ < 0.001
1856 reflections	Δρ_max_ = 0.22 e Å^−^^3^
136 parameters	Δρ_min_ = −0.16 e Å^−^^3^
Primary atom site location: structure-invariant direct methods	Extinction correction: none

### Special details

**Table:**

Geometry. All e.s.d.'s (except the e.s.d. in the dihedral angle between two l.s. planes) are estimated using the full covariance matrix. The cell e.s.d.'s are taken into account individually in the estimation of e.s.d.'s in distances, angles and torsion angles; correlations between e.s.d.'s in cell parameters are only used when they are defined by crystal symmetry. An approximate (isotropic) treatment of cell e.s.d.'s is used for estimating e.s.d.'s involving l.s. planes.
Refinement. Refinement of *F*^2^ against ALL reflections. The weighted *R*-factor *wR* and goodness of fit *S* are based on *F*^2^, conventional *R*-factors *R* are based on *F*, with *F* set to zero for negative *F*^2^. The threshold expression of *F*^2^ > σ(*F*^2^) is used only for calculating *R*-factors(gt) *etc*. and is not relevant to the choice of reflections for refinement. *R*-factors based on *F*^2^ are statistically about twice as large as those based on *F*, and *R*- factors based on ALL data will be even larger.

### Fractional atomic coordinates and isotropic or equivalent isotropic displacement parameters (Å^2^)

**Table:**

	*x*	*y*	*z*	*U*_iso_*/*U*_eq_	
N1	0.50902 (11)	0.26458 (10)	0.1311 (3)	0.0316 (5)	
N2	0.55431 (11)	0.19843 (11)	0.0995 (3)	0.0363 (5)	
N3	0.45643 (11)	0.51464 (11)	0.2591 (3)	0.0329 (5)	
H3A	0.4611	0.4662	0.2161	0.049*	
H3B	0.4679	0.5481	0.1675	0.049*	
H3C	0.4078	0.5226	0.2977	0.049*	
O1	0.50279 (10)	0.39457 (9)	0.0303 (2)	0.0428 (5)	
C1	0.53354 (13)	0.32631 (13)	0.0203 (3)	0.0320 (5)	
C2	0.59414 (14)	0.29704 (13)	−0.0881 (3)	0.0354 (6)	
H2	0.6225	0.3239	−0.1788	0.042*	
C3	0.60418 (14)	0.21958 (13)	−0.0345 (3)	0.0364 (6)	
C4	0.66173 (18)	0.16275 (17)	−0.1105 (4)	0.0579 (8)	
H4A	0.6533	0.1128	−0.0533	0.087*	
H4B	0.7135	0.1804	−0.0825	0.087*	
H4C	0.6554	0.1585	−0.2446	0.087*	
C5	0.46305 (12)	0.26868 (12)	0.2962 (3)	0.0298 (5)	
C6	0.39474 (13)	0.31290 (14)	0.2980 (3)	0.0373 (6)	
H6	0.3778	0.3380	0.1893	0.045*	
C7	0.35267 (15)	0.31879 (15)	0.4635 (4)	0.0444 (7)	
H7	0.3077	0.3489	0.4661	0.053*	
C8	0.37619 (15)	0.28079 (15)	0.6244 (4)	0.0451 (7)	
H8	0.3476	0.2857	0.7351	0.054*	
C9	0.44250 (15)	0.23530 (14)	0.6208 (4)	0.0414 (6)	
H9	0.4578	0.2085	0.7285	0.050*	
C10	0.48621 (13)	0.22946 (13)	0.4580 (3)	0.0349 (6)	
H10	0.5311	0.1993	0.4567	0.042*	
C11	0.51023 (13)	0.52621 (14)	0.4182 (3)	0.0349 (6)	
H11A	0.5080	0.5801	0.4588	0.042*	
H11B	0.5630	0.5153	0.3773	0.042*	

### Atomic displacement parameters (Å^2^)

**Table:**

	*U*^11^	*U*^22^	*U*^33^	*U*^12^	*U*^13^	*U*^23^
N1	0.0388 (11)	0.0248 (10)	0.0314 (10)	0.0026 (8)	0.0055 (9)	0.0014 (8)
N2	0.0446 (12)	0.0273 (10)	0.0370 (11)	0.0061 (9)	0.0070 (9)	0.0007 (8)
N3	0.0364 (11)	0.0309 (10)	0.0313 (10)	0.0010 (8)	0.0033 (8)	0.0021 (8)
O1	0.0609 (11)	0.0284 (9)	0.0391 (10)	0.0102 (8)	0.0137 (8)	0.0063 (7)
C1	0.0414 (13)	0.0280 (12)	0.0266 (11)	−0.0013 (10)	−0.0001 (10)	0.0003 (9)
C2	0.0432 (14)	0.0328 (13)	0.0300 (12)	0.0004 (11)	0.0083 (10)	0.0022 (10)
C3	0.0410 (14)	0.0358 (13)	0.0324 (13)	0.0055 (11)	0.0017 (11)	−0.0037 (10)
C4	0.070 (2)	0.0500 (17)	0.0532 (17)	0.0183 (15)	0.0204 (15)	0.0015 (14)
C5	0.0292 (12)	0.0264 (12)	0.0338 (12)	−0.0034 (9)	0.0014 (10)	0.0002 (9)
C6	0.0349 (13)	0.0371 (13)	0.0398 (14)	0.0024 (11)	−0.0013 (11)	0.0043 (11)
C7	0.0378 (14)	0.0427 (15)	0.0526 (16)	0.0072 (11)	0.0107 (12)	0.0049 (13)
C8	0.0450 (15)	0.0485 (16)	0.0417 (15)	−0.0008 (12)	0.0152 (12)	0.0036 (12)
C9	0.0452 (15)	0.0442 (15)	0.0349 (14)	−0.0008 (12)	0.0026 (11)	0.0083 (11)
C10	0.0334 (13)	0.0324 (13)	0.0388 (14)	0.0009 (10)	−0.0004 (11)	0.0020 (10)
C11	0.0321 (12)	0.0387 (14)	0.0340 (13)	−0.0020 (10)	−0.0003 (10)	0.0026 (10)

### Geometric parameters (Å, °)

**Table:**

N1—C1	1.385 (3)	C4—H4C	0.9600
N1—N2	1.395 (2)	C5—C10	1.388 (3)
N1—C5	1.414 (3)	C5—C6	1.398 (3)
N2—C3	1.330 (3)	C6—C7	1.382 (3)
N3—C11	1.472 (3)	C6—H6	0.9300
N3—H3A	0.8900	C7—C8	1.375 (4)
N3—H3B	0.8900	C7—H7	0.9300
N3—H3C	0.8900	C8—C9	1.382 (4)
O1—C1	1.288 (3)	C8—H8	0.9300
C1—C2	1.389 (3)	C9—C10	1.381 (3)
C2—C3	1.394 (3)	C9—H9	0.9300
C2—H2	0.9300	C10—H10	0.9300
C3—C4	1.490 (3)	C11—C11^i^	1.510 (4)
C4—H4A	0.9600	C11—H11A	0.9700
C4—H4B	0.9600	C11—H11B	0.9700
			
C1—N1—N2	111.27 (17)	C10—C5—C6	119.8 (2)
C1—N1—C5	126.97 (18)	C10—C5—N1	120.0 (2)
N2—N1—C5	119.01 (17)	C6—C5—N1	120.2 (2)
C3—N2—N1	104.56 (18)	C7—C6—C5	119.1 (2)
C11—N3—H3A	109.5	C7—C6—H6	120.5
C11—N3—H3B	109.5	C5—C6—H6	120.5
H3A—N3—H3B	109.5	C8—C7—C6	121.1 (2)
C11—N3—H3C	109.5	C8—C7—H7	119.5
H3A—N3—H3C	109.5	C6—C7—H7	119.5
H3B—N3—H3C	109.5	C7—C8—C9	119.7 (2)
O1—C1—N1	122.8 (2)	C7—C8—H8	120.2
O1—C1—C2	131.9 (2)	C9—C8—H8	120.2
N1—C1—C2	105.35 (19)	C10—C9—C8	120.3 (2)
C1—C2—C3	106.7 (2)	C10—C9—H9	119.8
C1—C2—H2	126.6	C8—C9—H9	119.8
C3—C2—H2	126.6	C9—C10—C5	120.0 (2)
N2—C3—C2	112.1 (2)	C9—C10—H10	120.0
N2—C3—C4	120.4 (2)	C5—C10—H10	120.0
C2—C3—C4	127.5 (2)	N3—C11—C11^i^	111.2 (2)
C3—C4—H4A	109.5	N3—C11—H11A	109.4
C3—C4—H4B	109.5	C11^i^—C11—H11A	109.4
H4A—C4—H4B	109.5	N3—C11—H11B	109.4
C3—C4—H4C	109.5	C11^i^—C11—H11B	109.4
H4A—C4—H4C	109.5	H11A—C11—H11B	108.0
H4B—C4—H4C	109.5		
			
C1—N1—N2—C3	−2.0 (2)	C1—N1—C5—C10	−130.8 (2)
C5—N1—N2—C3	−164.5 (2)	N2—N1—C5—C10	28.7 (3)
N2—N1—C1—O1	−177.0 (2)	C1—N1—C5—C6	48.4 (3)
C5—N1—C1—O1	−16.3 (4)	N2—N1—C5—C6	−152.1 (2)
N2—N1—C1—C2	2.0 (2)	C10—C5—C6—C7	2.1 (3)
C5—N1—C1—C2	162.8 (2)	N1—C5—C6—C7	−177.1 (2)
O1—C1—C2—C3	177.8 (2)	C5—C6—C7—C8	−1.2 (4)
N1—C1—C2—C3	−1.2 (3)	C6—C7—C8—C9	−0.6 (4)
N1—N2—C3—C2	1.3 (3)	C7—C8—C9—C10	1.6 (4)
N1—N2—C3—C4	−178.4 (2)	C8—C9—C10—C5	−0.8 (4)
C1—C2—C3—N2	−0.1 (3)	C6—C5—C10—C9	−1.1 (3)
C1—C2—C3—C4	179.6 (2)	N1—C5—C10—C9	178.1 (2)

Symmetry codes: (i) −*x*+1, −*y*+1, −*z*+1.

### Hydrogen-bond geometry (Å, °)

**Table:**

*D*—H···*A*	*D*—H	H···*A*	*D*···*A*	*D*—H···*A*
N3—H3A···O1	0.89	1.94	2.743 (2)	149
N3—H3B···O1^ii^	0.89	1.79	2.672 (2)	173
N3—H3C···N2^iii^	0.89	2.04	2.924 (3)	173

Symmetry codes: (ii) −*x*+1, −*y*+1, −*z*; (iii) −*y*+1/2, *x*, −*z*+1/2.
